# Optogenetics-based localization of talin to the plasma membrane promotes activation of β3 integrins

**DOI:** 10.1016/j.jbc.2021.100675

**Published:** 2021-04-16

**Authors:** Zhongji Liao, Alexandre R. Gingras, Frederic Lagarrigue, Mark H. Ginsberg, Sanford J. Shattil

**Affiliations:** Department of Medicine, University of California, San Diego, La Jolla, California, USA

**Keywords:** integrin, talin, platelet, endothelial cell, optogenetics, CHO, Chinese hamster ovary, CRY2, *Arabidopsis* cryptochrome 2, FERM, 4.1 protein/ezrin/radixin/moesin, HBSS, Hank's balanced salt solution, PAC-1, activation-dependent anti-αIIbβ3 monoclonal antibody, PHR, photolyase homology region, Rap1, Ras-related protein 1, sgRNA, single-guide RNA, THD, talin head domain

## Abstract

Interaction of talin with the cytoplasmic tails of integrin β triggers integrin activation, leading to an increase of integrin affinity/avidity for extracellular ligands. In talin KO mice, loss of talin interaction with platelet integrin αIIbβ3 causes a severe hemostatic defect, and loss of talin interaction with endothelial cell integrin αVβ3 affects angiogenesis. In normal cells, talin is autoinhibited and localized in the cytoplasm. Here, we used an optogenetic platform to assess whether recruitment of full-length talin to the plasma membrane was sufficient to induce integrin activation. A dimerization module (*Arabidopsis* cryptochrome 2 fused to the N terminus of talin; N-terminal of cryptochrome-interacting basic helix-loop-helix domain ended with a CAAX box protein [C: cysteine; A: aliphatic amino acid; X: any C-terminal amino acid]) responsive to 450 nm (blue) light was inserted into Chinese hamster ovary cells and endothelial cells also expressing αIIbβ3 or αVβ3, respectively. Thus, exposure of the cells to blue light caused a rapid and reversible recruitment of *Arabidopsis* cryptochrome 2–talin to the N-terminal of cryptochrome-interacting basic helix-loop-helix domain ended with a CAAX box protein [C: cysteine; A: aliphatic amino acid; X: any C-terminal amino acid]–decorated plasma membrane. This resulted in β3 integrin activation in both cell types, as well as increasing migration of the endothelial cells. However, membrane recruitment of talin was not sufficient for integrin activation, as membrane-associated Ras-related protein 1 (Rap1)–GTP was also required. Moreover, talin mutations that interfered with its direct binding to Rap1 abrogated β3 integrin activation. Altogether, these results define a role for the plasma membrane recruitment of talin in β3 integrin activation, and they suggest a nuanced sequence of events thereafter involving Rap1–GTP.

Integrin adhesion receptors are composed of an α and a β type I transmembrane subunits. Activation of integrins is a regulated process that controls their affinity for and binding to extracellular adhesive ligands, and it is required for many mammalian processes, including development, hemostasis, wound healing, and immunity (1, 2, 3). A key step in integrin activation is the binding of the cytoskeletal protein talin to the integrin β cytoplasmic tail (4). This process is exemplified in platelets and endothelial cells by the β3 integrins, αIIbβ3 and αVβ3, respectively (5, 6, 7). Although αIIbβ3 is required for platelet aggregation (8, 9), αVβ3 functions in endothelial cell migration and angiogenesis (10, 11).

Talin is a 270-kDa cytoplasmic protein with an N-terminal 4.1 protein/ezrin/radixin/moesin (FERM) domain and a C-terminal rod domain, the latter composed of amphipathic helical bundles (Fig. 1*A*) (12). The talin FERM domain differs from those of typical ezrin/radixin/moesin proteins in that it has an additional F0 domain N-terminal to the remaining F1, F2, and F3 subdomains. F1 contains an unstructured loop and a ubiquitin-like fold (13) and F3 harbors the primary β-integrin tail–binding site (4, 14). In unstimulated cells, talin is thought to be in an “autoinhibited” conformation within the cytoplasm and unassociated with the plasma membrane and its integrins. In platelets, the cytoplasmic localization of autoinhibited talin prevents “inside-out” αIIbβ3 activation, the resultant binding of extracellular fibrinogen to αIIbβ3, and platelet aggregation. A recent model derived from cryo-EM (15) is relevant to talin autoinhibition: the talin rod domain folds into a 15-nm globular conformation that shields the binding site for integrin β tails in FERM F3 through interactions of rod domains R12 with F2 and R9 with F3, respectively (Fig. 1*A*). Thus, autoinhibited talin should have limited access to the plasma membrane and to integrins. However, when platelets are activated by one or more agonists such as ADP or thrombin (16), talin is recruited to the plasma membrane (17), presumably facilitated by the relief of talin autoinhibition as well as by talin interaction with membrane phospholipids *via* the flexible loop in FERM F1 (13, 18) and by basic residues in FERM F2/F3 (19). Indeed, the ability of talin to bind to membrane phospholipids and to activate integrins is reduced by deletion of the lipid-binding helix in talin F1 (13) or by specific mutations in F2 or F3 (19).

**Figure 1:**
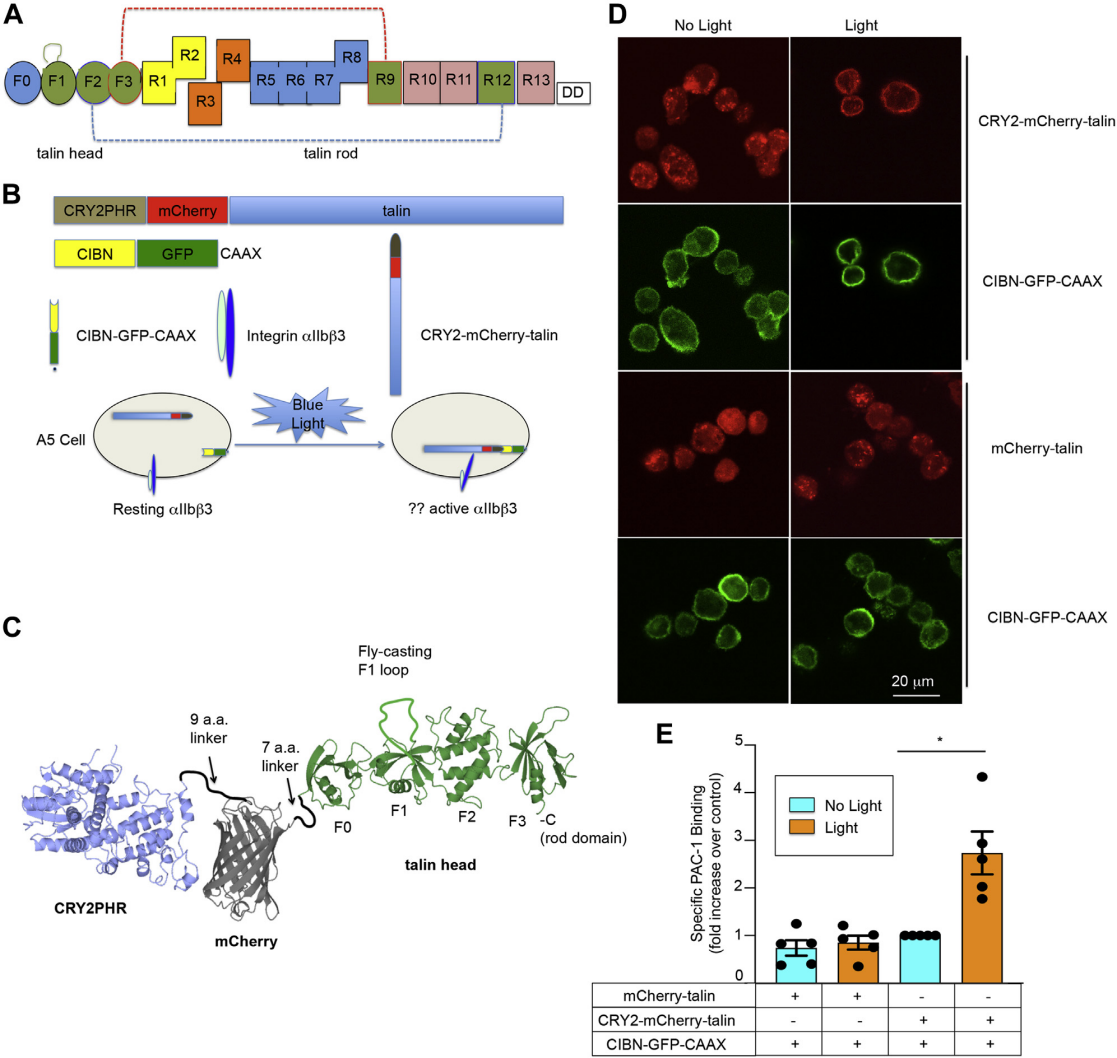
**Optogenetic recruitment of talin to the plasma membrane leads to activation of integrin αIIbβ3.***A*, linear depiction of talin domains. In a structural model of “autoinhibited” talin (15), the interactions between rod domain R9 and FERM subdomain F3 (*red dashed line*) and R12 and F2 (*blue dashed line*) limit F3 domain access to plasma membrane lipids and integrin β tails. Talin F1 contains a unique amphipathic helix loop that may facilitate talin interaction with the plasma membrane. *B*, depiction of the CIBN–GFP–CAAX and CRY2–mCherry–talin fusion proteins that were stably expressed in A5 CHO cells. CIBN–GFP–CAAX is constitutively anchored to the plasma membrane. As a result, when the cells are exposed to 450-nm (*blue*) light, the CIBN moiety should interact with CRY2, resulting in talin recruitment to the plasma membrane. *C*, a model of CRY2–mCherry–talin using atomic coordinates of each protein (CRY2PHR, mCherry, and talin head domain (THD)). The view highlights CRY2PHR (shown in *blue*), mCherry (shown in *gray*), and the F0, F1, F2, and F3 subdomains of talin (shown in *green*). For simplicity, the talin rod domain is not shown. Membrane translocation of CRY2–mCherry–talin was observed by confocal microscopy (panel *D*, *top*) in experiments where cultured cells were first trypsinized, suspended in buffer, and then exposed to pulsed blue light for 30 min with a frequency of 1 s each 75 s. No such membrane recruitment of talin was observed if cells expressed CIBN–GFP–CAAX and mCherry-talin (without CRY2) (panel *D*, *bottom*). The scale bar represents 20 μm. *E*, optogenetic recruitment of talin to the plasma membrane promotes αIIbβ3 activation, as monitored by specific PAC-1 binding quantified by flow cytometry. PAC-1 binding is expressed as the fold increase in binding relative to that observed in the same cells maintained in the dark. In this and subsequent figures, unless specified otherwise, PAC-1 binding was normalized to that observed when cells expressing CIBN–GFP–CAAX and CRY2–mCherry–WT talin were maintained in the dark. Data represent the means ± SEM of five experiments (*asterisk*, *p* < 0.05). CHO, Chinese hamster ovary; CRY2, *Arabidopsis* cryptochrome 2; FERM, 4.1 protein/ezrin/radixin/moesin; PAC-1, activation-dependent anti-αIIbβ3 monoclonal antibody; PHR, photolyase homology region.

αIIbβ3 activation in platelets requires agonist-dependent conversion of the membrane-anchored GTPase, Ras-related protein 1 (Rap1), from inactive Rap1–GDP to active Rap1–GTP (20, 21, 22). Recent work indicates that the relevant Rap1 effector for αIIbβ3 activation in platelets is talin itself because Rap1–GTP can interact directly with the talin F0 and F1 FERM subdomains (13, 18, 23) that may be solvent-accessible even in the autoinhibited conformation of talin (15, 24). Indeed, mutation of the Rap1 binding sites in talin profoundly suppresses αIIbβ3 activation in murine platelets (25). Because binding sites for phosphatidylinositol 4,5-bisphosphate [PtdIns (4,5)P2] in the F2 and F3 domains of talin are inaccessible in the autoinhibited full-length molecule, several research groups have proposed the hypothesis that Rap1 binding to the sites in F0 and F1 helps localize talin to the plasma membrane where PtdIns (4,5)P2 binding can uncover the integrin binding site in F3, resulting in integrin activation (15, 26, 27). Key unresolved issues in the process of integrin activation include the precise roles of initial talin recruitment to the plasma membrane and the molecular events that follow the interaction of talin with Rap1–GTP.

To begin to explore these questions, we have designed an optogenetic system to rapidly and reversibly enforce the recruitment of full-length talin to the plasma membranes of cells expressing αIIbβ3 or αVβ3. The results establish that talin recruitment to the plasma membrane is necessary, but not sufficient, for β3 integrin activation and function. Rather, integrin activation and adhesive function require additional events at the plasma membrane that are triggered by Rap1.

## Results and discussion

### Optogenetic recruitment of talin to the plasma membrane leads to activation of integrin αIIbβ3

To enable optogenetic recruitment of full-length talin to the plasma membrane, we stably expressed a pair of light-dependent dimerization modules in A5 Chinese hamster ovary (CHO) cells that express αIIbβ3 (Fig. 1*B*): (1) CIBN (a truncated version of CIB1 that is by itself deficient in homo-oligomerization and DNA binding) was fused to a short prenylated version of enhanced green fluorescent protein that terminates with a consensus CAAX sequence from Kirsten rat sarcoma for plasma membrane localization (28, 29, 30), and (2) a *Arabidopsis* cryptochrome 2 (CRY2) photolyase homology region (PHR) domain was fused to the N terminus of mCherry–talin. Juxtaposition of CRY2 to mCherry limits CRY2 homo-oligomerization (31). We modeled the structure of CRY2, mCherry, and talin using the atomic coordinates of each protein and found that the linkers we constructed between each protein would enable sufficient freedom of movement so that CRY2 and talin could interact properly with relevant components on the plasma membrane (Fig. 1*C*). Although CIBN–GFP–CAAX was constitutively associated with the plasma membrane of A5 cells, CRY2–mCherry–talin associated with the plasma membrane in response to 450-nm blue light (Fig. 1*D* and Fig. S1). Recruitment of talin to the plasma membrane could be observed within 1 min of blue light illumination and was reversible after elimination of the light (Fig. S2).

To investigate the functional consequences of optogenetic recruitment of talin to the plasma membrane, αIIbβ3 activation state was monitored by the binding of mAb, activation-dependent anti-αIIbβ3 monoclonal antibody (PAC-1), to the A5 CHO cells (32, 33). Previous work indicated that PAC-1 binding reports predominantly on affinity modulation of αIIbβ3 in these cells (34). In A5 cells co-expressing CIBN–GFP–CAAX and CRY2–mCherry–talin, specific PAC-1 binding increased significantly after blue light illumination (*p* < 0.05) (Fig. 1*E* and Fig. S3*A*). In contrast, neither talin translocation to the plasma membrane nor increased PAC-1 binding was observed if mCherry–talin lacking the CRY2 module was used (Fig. 1, *D* and *E* and Fig. S3*B*). The binding of fluorophore-conjugated PAC-1 triggered by blue light could be blocked by unlabeled, monovalent PAC-1 Fab (35), consistent with an effect of light-induced membrane recruitment of CRY2–mCherry–talin on αIIbβ3 affinity (Fig. S4*A*). In addition, membrane recruitment of talin promoted αIIbβ3 interaction with its physiological ligand, fibrinogen (Fig. S4*B*). These results indicate that optogenetic translocation of talin to the plasma membrane can lead to activation of αIIbβ3.

### Is membrane recruitment of talin, *per se*, sufficient for induction of αIIbβ3 activation?

**Figure 2:**
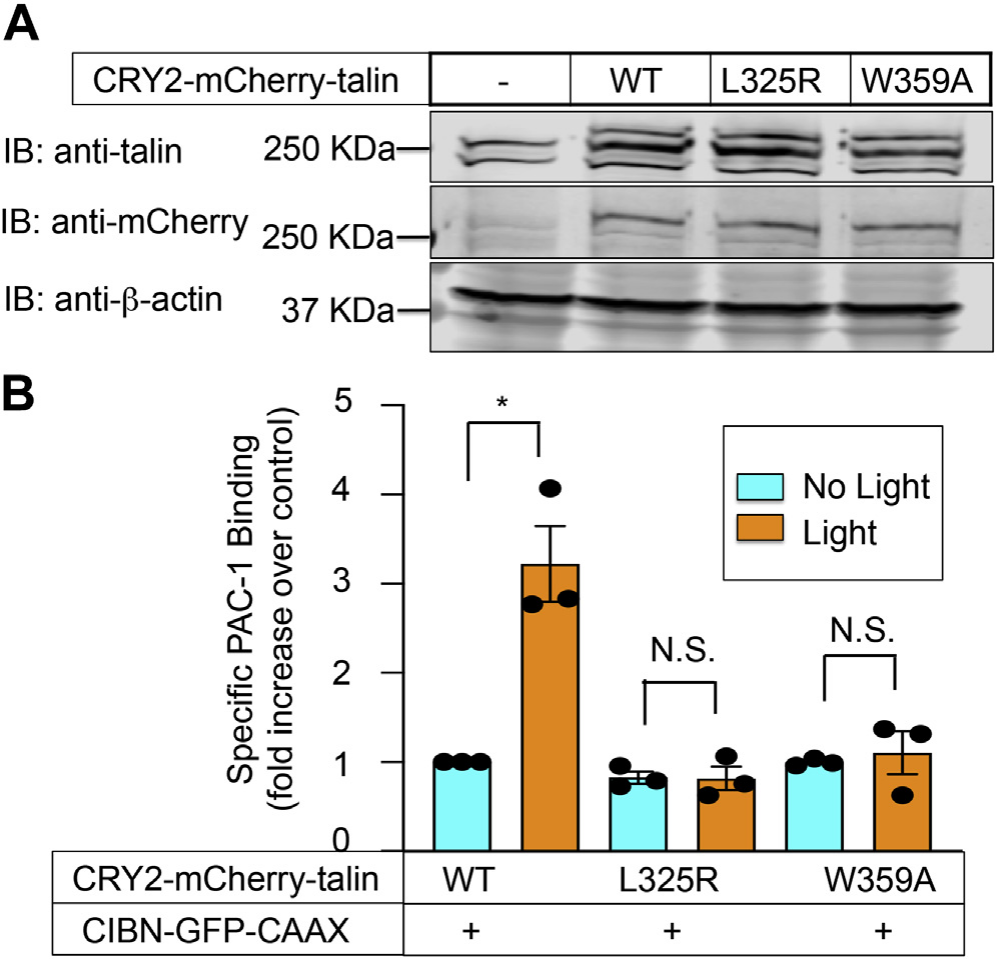
**Activation of αIIbβ3 after talin recruitment to the plasma membrane requires talin binding to the β3 cytoplasmic tail.***A*, A5 CHO cells were prepared that stably coexpressed C1BN–GFP–CAAX and either CRY2–mCherry–WT talin or one of two CRY2–mCherry–talin constructs (L325R or W359A) known to abrogate talin interaction with the β3 cytoplasmic tail (14). Talin expression was assessed by Western blot using antibodies to talin or to mCherry. β-actin was monitored as a loading control. Note that in the anti-talin antibody blot, the upper region of three immunoreactive bands represents CRY2–mCherry–talin, which was also immunoreactive with the anti-mCherry antibody. *B*, optogenetic recruitment of integrin β3 tail binding–deficient CRY2–mCherry–talin to the plasma membrane fails to activate αIIbβ3. Data represent the means ± SEM of three experiments (*asterisk*, *p* < 0.05). CHO, Chinese hamster ovary; CRY2, *Arabidopsis* cryptochrome 2; N.S., not statistically significant.

Structure–function studies of the talin FERM domain suggest that integrin activation requires the simultaneous binding of the F1/F2 subdomains to membrane microdomains enriched in PtdIns (4,5)P2 and the F3 subdomain to the integrin β cytoplasmic tail (4, 24, 36). To study this idea further using optogenetics, either of two F3 talin mutants (L325R or W359A) that disrupt talin interaction with integrin β tails (14) was introduced into CRY2–mCherry–talin. After the expression of either of these talin mutants in A5 CHO cells, their translocation to the plasma membrane in response to blue light was similar to that observed with CRY2–mCherry–WT talin (Fig. 2*A* and Fig. S1). On the other hand, neither mutant could activate αIIbβ3 upon recruitment to the plasma membrane (Fig. 2*B*). Thus, recruitment of talin to the plasma membrane may be necessary, but it is not sufficient for induction of αIIbβ3 activation. Rather, and as anticipated, binding of talin to the integrin β3 cytoplasmic tail is also required.

**Figure 3:**
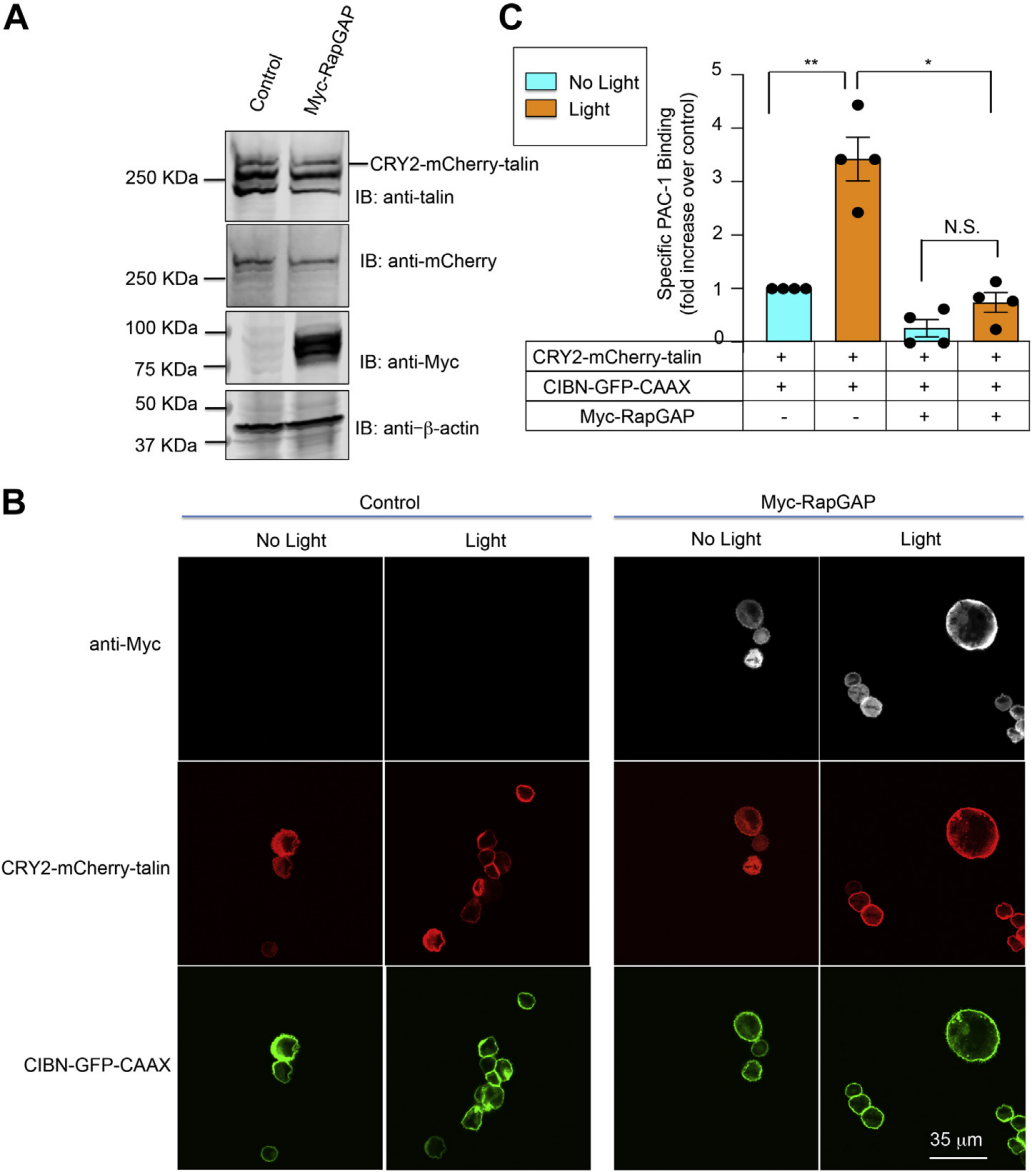
**Rap1 activity is required for αIIbβ3 activation after talin recruitment to the plasma membrane.** Lentivirus encoding Myc-tagged RapGAP was transduced into A5 cells stably expressing CRY2–mCherry–talin and CIBN–GFP–CAAX. Empty lentiviral vector was used as a control. *A*, expression of CRY2–mCherry–talin and Myc–RapGAP in the A5 cells was examined by Western blotting. β-Actin was monitored as a loading control. *B*, expression of RapGAP has no discernable effect on the optogenetic recruitment of talin to the plasma membrane as assessed by confocal microscopy. The scale bar represents 35 μm. *C*, RapGAP blocks activation of αIIbβ3 in response to optogenetic recruitment of talin to the plasma membrane. Data represent the means ± SEM of four experiments (*double asterisk*, *p* < 0.01; *asterisk*, *p* < 0.05). CRY2, *Arabidopsis* cryptochrome 2; N.S., not statistically significant; Rap1, Ras-related protein 1.

In platelets, agonist-induced αIIbβ3 activation requires activation of Rap1 (22), and talin is a direct target and effector of Rap1 in these cells (25, 26, 27). Consequently, to evaluate a potential role for Rap1 in αIIbβ3 activation after optogenetic recruitment of talin to the plasma membrane, A5 cells expressing CRY2–mCherry–talin and CIBN–GFP–CAAX were stably transduced with lentivirus encoding Myc-tagged Rap1GAP, which inhibits Rap1 activity by converting Rap1–GTP to Rap1–GDP (37). Although Rap1GAP did not affect the cellular expression of CRY2–mCherry–talin or its optogenetic recruitment to the plasma membrane (Fig. 3, *A* and *B*), it did prevent light-induced αIIbβ3 activation (Fig. 3*C*). Thus, in platelets where Rap1 is expected to be anchored to the plasma membrane through its own CAAX box motif (38), the generation of Rap1–GTP in response to platelet agonists may lead to αIIbβ3 activation through mechanism(s) that either precede and/or follow talin recruitment to the plasma membrane.

### Why is Rap1 needed for αIIbβ3 activation after talin recruitment to the plasma membrane?

**Figure 4:**
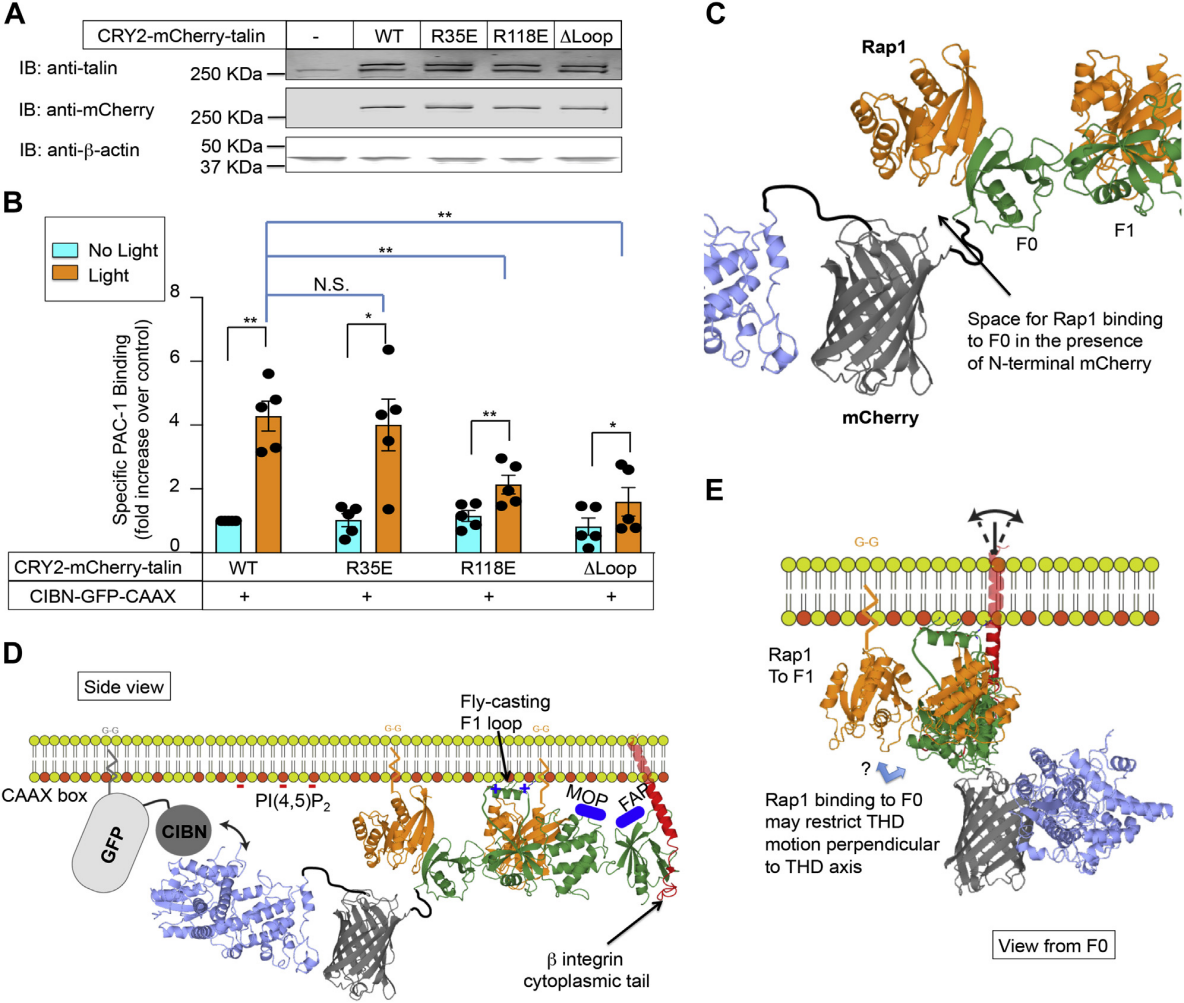
**αIIbβ3 activation requires direct interaction between talin and Rap1 after optogenetic recruitment of talin to the plasma membrane.***A*, talin mutations R35E, R118E, or ΔLoop were introduced into CRY2–mCherry–talin and the latter stably expressed along with C1BN–GFP–CAAX in A5 cells. Expression of CRY2–mCherry–talin was examined using anti-talin and anti-mCherry antibodies. β-Actin was monitored as a loading control. *B*, cells were treated with or without *blue* light and αIIbβ3 activation was assessed by specific PAC-1 binding. Data represent the means ± SEM of five experiments (*double asterisk*, *p* < 0.01; *asterisk*, *p* < 0.05). *C*, model of the CRY2–mCherry–talin head domain (THD) F0/F1 region. Note that two binding sites for Rap1–GTP (shown in *orange*) are potentially available within F0 and F1 subdomains of CRY2–mCherry–talin. *D*, view of CRY2–mCherry–THD as seen from the angle of the plasma membrane where CRY2 binds to CIBN–GFP–CAAX. This model summarizes the interactions of THD at the plasma membrane. Negatively charged PtdIns(4,5)P2 is shown as *red dots*; two Rap1 molecules are depicted in *orange*; the integrin β-tail traversing the plasma membrane is depicted in *red*. The structure of CIBN is not available, so it is drawn to scale according to its molecular weight. Both Rap1–GTP and CIBN–GFP–CAAX are membrane-associated *via* their C-terminal geranyl–geranyl (G–G) moieties. Upon contact with Rap1 and negatively charged phospholipids, the F1 loop in the THD is modeled to switch into the helical state, resulting in a cluster of positive charges to interact with plasma membrane. In addition, F2–F3 subdomains contain regions that face the membrane and interact with negatively charged phospholipids. They include the F2 membrane orientation patch (MOP) and the F3 association patch (FAP) (both shown in *blue*). *E*, view of CRY2–mCherry–THD as seen from the angle of the F0 subdomain on the plasma membrane. Note that the position of Rap1 bound to the F1 subdomain is perpendicular to the THD axis. CRY2, *Arabidopsis* cryptochrome 2; N.S., not statistically significant; PAC-1, activation-dependent anti-αIIbβ3 monoclonal antibody; PtdIns(4,5)P2, phosphatidylinositol 4,5-bisphosphate; Rap1, Ras-related protein 1.

Because Rap1–GTP can interact directly with talin (18, 23, 39), we posited that it functions to appropriately localize and optimally orient talin with respect to the β3 cytoplasmic tail. In this regard, the loss of low-affinity binding of the talin F0 subdomain to Rap1 has only a modest effect on αIIbβ3 activation in murine platelets (26, 27). On the other hand, Rap1–GTP binding to the talin F1 subdomain and a unique loop in F1 that has a propensity to form a helix upon binding to membrane lipids work in tandem to enable direct and productive Rap1–talin interaction, resulting in αIIbβ3 activation, both in A5 CHO cells (18) and platelets (see Fig. 1, *A* and *C*) (25). Therefore, we introduced disruptive mutations into the relevant loci of F0 (R35E) or F1 (R118E) within CRY2–mCherry–talin (13, 18). In A5 cells, neither mutation adversely affected CRY2–mCherry–talin expression or its optogenetic recruitment to the plasma membrane (Fig. 4*A* and Fig. S1). However, while CRY2–mCherry–talin (R35E) retained the ability to activate αIIbβ3 in response to blue light, the ability of CRY2–mCherry–talin (R118E) to activate αIIbβ3 was significantly impaired (*p* < 0.01) (Fig. 4*B*). Furthermore, when the membrane-binding loop in F1 was deleted from CRY2–mCherry–talin, αIIbβ3 activation in response to blue light was similarly impaired (Fig. 4*B*).

These results suggest that one function of Rap1 binding to talin F1 in tandem with the F1 loop is to promote the correct membrane localization and orientation of talin with respect to αIIbβ3. Indeed, in our atomic model of CRY2–mCherry–talin, there would appear to be adequate space for Rap1 to interact with its interaction sites in the F0 and F1 FERM subdomains of talin (Fig. 4*C*). Consequently, we modeled the talin head domain (THD) of CRY2–mCherry–talin in the correct orientation to enable CRY2 interaction with the N terminus of CIBN–GFP–CAAX in response to blue light, and simultaneously with two Rap1 molecules, negatively charged plasma membrane phospholipids, and the integrin β3 cytoplasmic tail (Fig. 4*D*). The model suggests that the flexible loop in talin F1 and its Rap1-binding sites may place the THD in the correct orientation for interaction with the β3 cytoplasmic tail. Specifically, the F1 binding site in the THD for Rap1 may be located perpendicular to the axis of the THD, thus imparting the THD with the right tilting angle to the β3 integrin transmembrane domain to activate the integrin (Fig. 4*E*).

**Figure 5:**
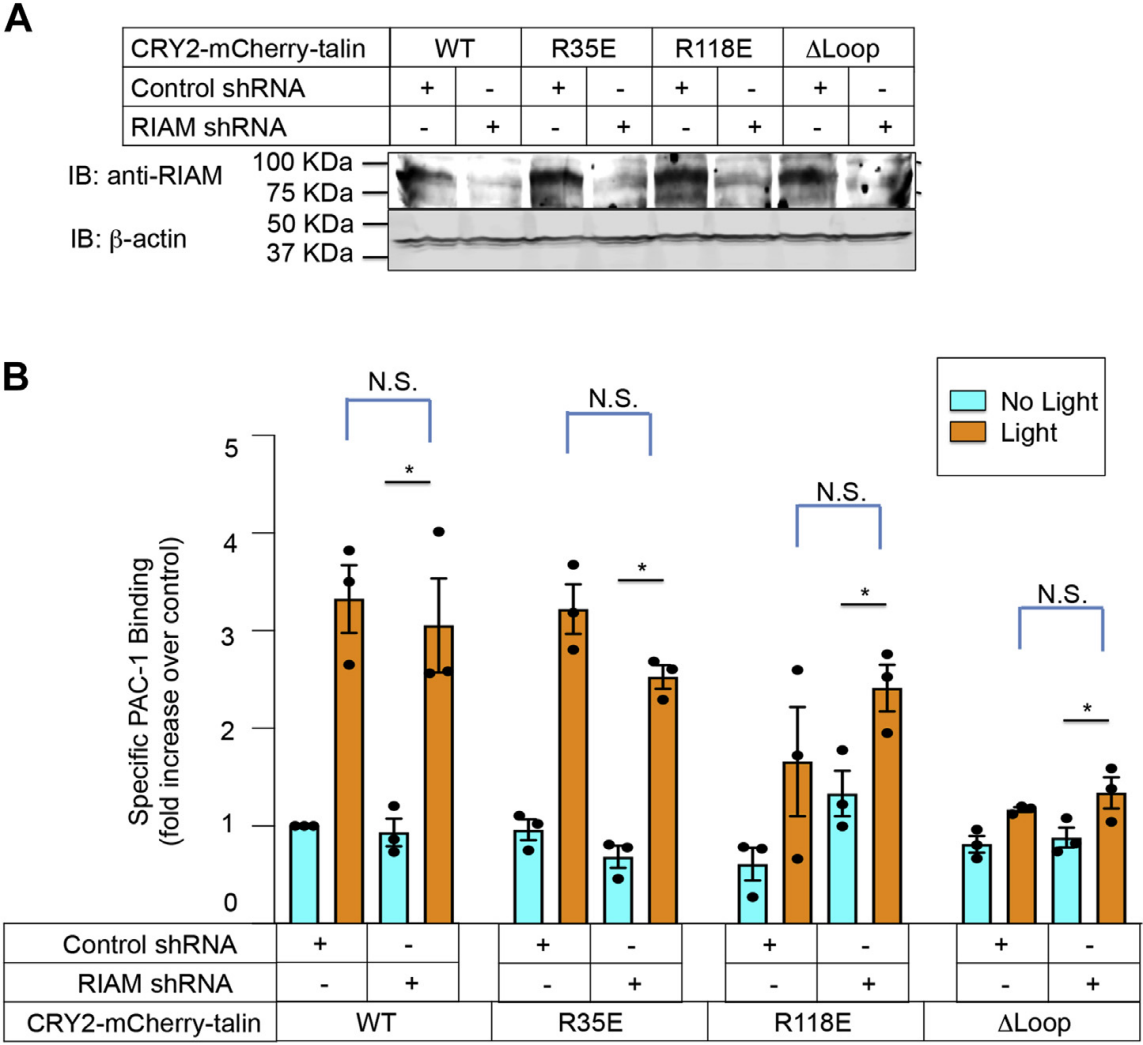
**Activation of αIIbβ3 by plasma membrane–associated talin does not require RIAM.** Lentivirus encoding RIAM shRNA or control shRNA was transduced into A5 cells expressing C1BN–GFP–CAAX and one of the CRY2–mCherry–talin constructs as indicated. *A*, RIAM expression was assessed on Western blots using an anti-RIAM antibody. β-Actin was monitored as a loading control. Note the knockdown of RIAM in each of the cell lines by RIAM shRNA. *B*, αIIbβ3 activation was monitored with PAC-1 expressed as the fold increase in binding relative to binding in control shRNA-transduced cells maintained in the dark. Data represent the means ± SEM of three experiments (*asterisk*, *p* < 0.05). CRY2, *Arabidopsis* cryptochrome 2; N.S., not statistically significant; PAC-1, activation-dependent anti-αIIbβ3 monoclonal antibody; RIAM, Rap1-GFP-interacting adaptor molecule.

In leukocytes, Rap1 regulates the interaction of talin with β2 integrins through the Rap1 effector, Rap1-GFP-interacting adaptor molecule (RIAM) (3, 40). However, expression of RIAM is low in platelets and dispensable for αIIbβ3 activation (41, 42, 43). Consistent with those results, RIAM knockdown by shRNA in A5 cells failed to inhibit the optogenetic activation of αIIbβ3 after membrane recruitment of CRY2–mCherry–talin (Fig. 5). These results reinforce the idea (18, 23, 39) that talin itself is the relevant Rap1 effector for αIIbβ3 activation.

A recent crystal structure of the talin 1 FERM domain, adopting the canonical cloverleaf shape, was solved (44). In this model, both the F0 and F1 domains are positioned away from the F2–F3 domains and the plasma membrane to allow binding of PI(4,5)P2 at the F2–F3 interface (19) and binding of the integrin β tail to F3 (4). However, further investigation is required to determine how the FERM-folded model might accommodate with the direct Rap1 binding within F0/F1. It is possible that the F1–F2 linker is flexible, as observed in small-angle X-ray scattering data on the talin 1 head domain (24), as well as in the cryo-EM structure of talin 1 (15) and the crystal structure of talin 2 (45). One alternative idea would be that the folded FERM works when RIAM or Lamellipodin functions as Rap1 effectors to recruit talin at the plasma membrane. However, in the present study, we show that RIAM knockdown by shRNA in A5 cells failed to inhibit the optogenetic activation of αIIbβ3 and thus direct Rap1 binding to the talin F1 domain is required (Fig. 5).

### Optogenetic recruitment of talin to the endothelial cell plasma membrane leads to activation of integrin αVβ3

**Figure 6:**
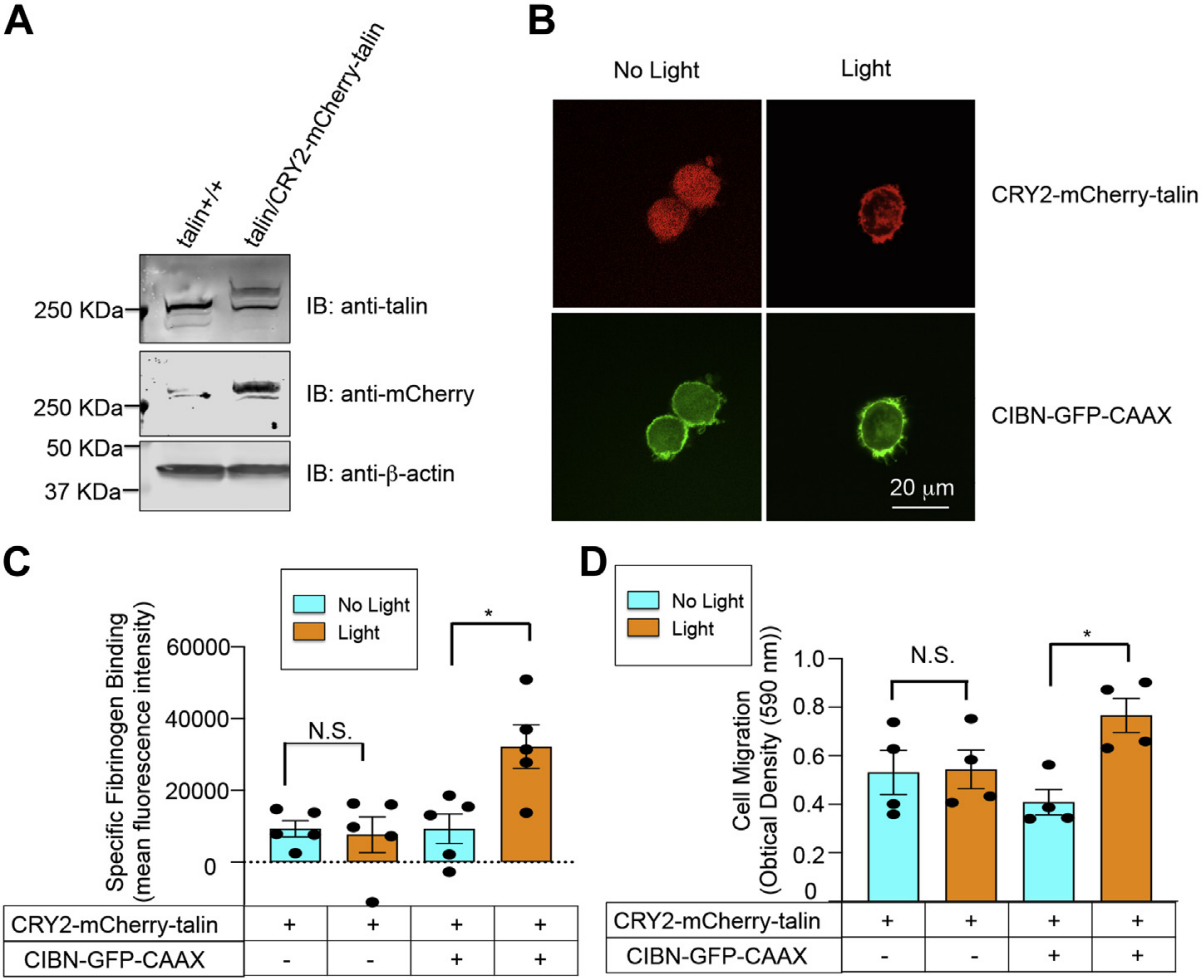
**Optogenetic recruitment of talin to the plasma membrane activates integrin αVβ3 in endothelial cells.** CRISPR–Cas9 was utilized to “tag” endogenous talin in endothelial cells with CRY2–mCherry, as detailed in Experimental procedures. Cells also stably expressed CIBN–GFP–CAAX. *A*, Western blots showing the expression of CRY2–mCherry–talin using anti-talin and anti-mCherry antibodies. Parental immortalized mouse lung endothelial cells were used as a negative control and β-actin as a loading control. *B*, recruitment of CRY2–mCherry–talin to the plasma membrane of endothelial cells maintained in suspension and exposed to 450-nm *blue* light for 30 min. The scale bar represents 20 μm. *C*, optogenetic recruitment of talin to endothelial cell plasma membranes induces fibrinogen binding to integrin αVβ3. Specific fibrinogen binding was measured by flow cytometry as described in Experimental procedures. Data represent the means ± SEM of five experiments (*asterisk*, *p* < 0.05). *D*, optogenetic recruitment of talin to the plasma membrane promotes endothelial cell migration. Cell migration across fibrinogen-coated transwells was determined as described in Experimental procedures. Data represent the means ± SEM of four experiments (*asterisk*, *p* < 0.05). CRY2, *Arabidopsis* cryptochrome 2; N.S., not statistically significant.

To determine the effects of membrane recruitment of talin in a vascular cell endogenously expressing integrin αVβ3, optogenetic experiments were performed in immortalized murine lung microvascular endothelial cells. Full-length talin in these cells was conjugated at the N terminus to CRY2–mCherry utilizing CRISPR–Cas9–based homology-directed repair (Fig. 6*A* and Fig. S5). After the stable expression of CIBN–GFP–CAAX in these cells, optogenetic recruitment of CRY2–mCherry–talin to the plasma membrane resulted in ∼3-fold increase in specific binding of soluble fibrinogen, whereas cells expressing CRY2–mCherry–talin but not CIBN–GFP–CAAX showed no such response (Fig. 6, *B* and *C*). Furthermore, optogenetic recruitment of talin to the plasma membrane increased endothelial cell migration across fibrinogen-coated transwells (Fig. 6*D*).

Collectively, these results help define events involved in talin-dependent β3 integrin activation in cells, and they illuminate nuances to the process. We show that recruitment of talin to the plasma membrane represents an important but not sufficient step in the sequence of events. In cells such as platelets, agonists such as ADP and thrombin are required to initiate the process by activating CalDAG–GEF1 and converting membrane-associated Rap1–GDP to Rap1–GTP (20, 21, 22). Platelet activation may also lead to additional biochemical events that facilitate relief of talin autoinhibition. Our data show that in addition to likely facilitating membrane recruitment of talin in primary cells such as platelets and endothelial cells, the direct interaction of talin with Rap1–GTP can place talin in proximity to and in appropriate orientation with plasma membrane phospholipids and the integrin β3 cytoplasmic tail, leading to integrin activation. In addition, kindlin, a known integrin coactivator, may function in part by enhancing talin recruitment to the plasma membrane in proximity to β3 integrins (46) and/or by promoting integrin clustering (47, 48). As A5 CHO cells and murine lung microvascular endothelial cells express the kindlin-2 isoform, these cells may also provide tractable optogenetic platforms for further studies of kindlin function in integrin biology.

## Experimental procedures

### Materials and DNA constructs

Alexa Fluor 647–conjugated PAC-1, an activation-dependent anti-αIIbβ3 mAb (32), was provided by BioLegend. PAC-1 Fab fragment, activation-independent anti-αIIbβ3 antibody D57, and αIIbβ3-activating antibody Ab33 were described previously (35, 40, 49). Alexa Fluor 647–conjugated fibrinogen was from Thermo Fisher Scientific. Rabbit polyclonal anti-mCherry/DsRed antibody was from Takara. Mouse monoclonal anti-talin and anti-β-actin antibodies were from Sigma-Aldrich. Rabbit polyclonal antibody against RIAM was described previously (50).

pCIBN(deltaNLS)–pmGFP (plasmid 26867) and pCRY2PHR–mCherryN1 (plasmid 26866) (51) were obtained from Addgene. To generate CRY2–mCherry–talin, PCR-amplified CRY2PHR–mCherry and full-length murine talin including an N-terminal seven amino acid linker (EFAEAAT) were inserted into EcoRI and BamHI sites of pcDNA3.1 Zeo(+) using the In-Fusion cloning kit (Takara). PCR-amplified CIBN–GFP–CAAX from pCIBN(deltaNLS)–pmGFP were inserted into EcoRI and BamHI sites of vector pLVX-het1. Mutations in CRY2–mCherry–talin were introduced using the KAPA HiFi kit (Kapa Biosystems). Rap1GAP was cloned into XhoI and BamHI sites of pLVX-het2. Lentiviral constructs in FG12 encoding RIAM shRNA or control shRNAs were described previously (40, 52). Mouse talin single-guide RNA (sgRNA) (5’-GCTTCAGCGAAAGCGCAACCA-3’) was constructed in vector pSpCas9(BB)-2A-GFP (48140; Addgene) (53). The sequence encoding CRY2–mCherry was cloned into a homology-directed repair template plasmid between the sequences of the talin promoter to talin exon 2 and talin exons 2 to 6.

### Cell culture and transfection

A5 CHO cells stably expressing αIIbβ3 (54) were cultured in Dulbecco's modified Eagle's medium supplemented with nonessential amino acids, antibiotics, L-glutamine, and 10% fetal bovine serum. TransIT-X2 (Mirus Bio) or Lipofectamine 2000 (Thermo Fisher Scientific) was used for transfection, according to the manufacturers’ protocols. A5 cells stably expressing C1BN–GFP–CAAX were generated by lentiviral transduction (11). Cells were then transfected with plasmids pcDNA3.1 Zeo(+) encoding CRY2–mCherry–talin, followed by selection with 250 μg/ml Zeocin. Cells double-positive for GFP and mCherry were sorted by flow cytometry on a FACSAria (BD Biosciences).

To generate endothelial cells stably expressing CRY2–mCherry–talin, immortalized mouse lung endothelial cells (11) were cotransfected with pSpCas9(BB)–2A–GFP containing mouse talin sgRNA and a homology-directed repair template plasmid for CRY2–mCherry using the TransIT–X2 reagent. CRY2–mCherry (∼2.2 Kbp) was flanked by the sequences of the talin promoter to talin exon 2 (∼1.4 Kbp) and talin exon 2 to exon 6 (∼1.4 Kbp) (Fig. S5*A*). Two days after transfection, GFP-positive cells were sorted and cultured as described (11). Two weeks after culture, single cells positive for mCherry were sorted by flow cytometry into 96-well plates. In total, 21 clones survived and five were verified positive for mCherry by fluorescence microscopy. DNA was extracted from two of the cell lines, and the sgRNA-targeted region was amplified using PCR (Fig. S6*B*) and sequenced to confirm CRY2–mCherry tagging of talin.

### Confocal microscopy and image analysis

Cells were harvested with trypsin and suspended in Hank's balanced salt solution (HBSS) buffer containing calcium and magnesium (Gibco, Thermo Fisher Scientific) supplemented with 1% (wt/vol) bovine serum albumin (Sigma-Aldrich). Cells were then illuminated using a blue light–emitting diode (LED) mounted in a customized chamber (1 s illumination per 75 s; 50 mW/cm^2^) for 30 min before fixation with 3.7% formaldehyde. After washing with PBS, coverslips were mounted on slides using the ProLong Diamond antifade reagent (Thermo Fisher Scientific). Images were taken with an Olympus IX81 inverted microscope, using a 100X oil objective (numerical aperture 1.4). For qualification of membrane recruitment of CRY2–mCherry–talin, GFP- and mCherry-positive pixels were subjected to background correction. Intensities of mCherry were measured using Volocity (PerkinElmer) on subareas at cell edges where GFP-positive pixels were selected from 10 to 15 cells in each of two independent experiments.

### Integrin activation

Integrin αIIbβ3 activation in CHO cells was assessed by flow cytometry with Alexa Fluor 647–conjugated antibody PAC-1 (32, 33). Briefly, cells were trypsinized and suspended in the HBSS buffer described above. 50 μl of cell suspension were incubated with 2 μl of Alexa Fluor 647–conjugated PAC-1 and illuminated using blue LED (1 s illumination per 75 s; 50 mW/cm^2^) for 30 min at room temperature (RT) before fixation with 3.7% formaldehyde. To detect fibrinogen binding to CHO cells or to immortalized mouse endothelial cells, 4 to 6 × 10^5^ cells in 50-μl HBSS containing 1% bovine serum albumin were incubated with Alexa Fluor 647–conjugated fibrinogen and illuminated using blue LED for 30 min at RT before fixation with 3.7% formaldehyde. Nonspecific PAC-1 or fibrinogen binding was determined in the presence of 5 mM EDTA, and specific binding was taken as total minus nonspecific binding. β3 activating antibody Ab33 was used at 150 μg/ml.

### Cell migration

Transwells with 8-μm pores were coated with 100 μg/ml fibrinogen. Then, 10^5^ endothelial cells in 200 μl of the endothelial basal medium were added to the upper chamber, and 700 μl of the medium containing 2% fetal bovine serum was added to the lower chamber. Cells were incubated for 16 h at 37 °C and 5% CO_2_ with or without LED illumination from the top of the transwells. Cells on the upper surface of the chamber were removed with a cotton swab, and migrated cells on the lower surface were fixed with 4% paraformaldehyde for 15 min and stained with 0.1% crystal violet. Crystal violet staining was eluted in 10% acetic acid, and absorbance was measured at 590 nm.

### Western blotting

Cells were lysed in NP-40 buffer (1% Nonidet P-40, 150 mM NaCl, 50 mM Tris HCl, pH 8.0, and EDTA-free complete protease inhibitor cocktail (Roche Applied Science)). Proteins were resolved by SDS-PAGE and transferred to nitrocellulose membranes. After blocking with 5% bovine serum albumin in Tris-buffered saline, membranes were incubated with appropriate primary and secondary antibodies, the latter conjugated to IRDye 800CW or IRDye 680RD. Blots were analyzed with the Odyssey imaging system (LI-COR Biosciences). Full western blots are displayed in Fig. S7.

### Statistical analysis

Paired, two-tailed Student's *t* test was used to calculate differences between two groups. *p* values of ≤0.05 were taken as statistically significant.

## Data availability

All data are included in the article and the Supporting information.

## Supporting information

This article contains supporting information.

## Conflict of interest

The authors declare that they have no conflicts of interest with the contents of this article.
