# Supplementary material for: Optogenetics-based localization of talin to the plasma membrane promotes activation of β3 integrins
Source: J Biol Chem. 2021 Apr 16;296:100675. doi: 10.1016/j.jbc.2021.100675 (PMC8131925; doi:10.1016/j.jbc.2021.100675)
Supplement: Supplemental Figures S1–S7 [file mmc1.pdf]

Supplementary Information for

Optogenetic-based Localization of Talin to the Plasma Membrane Promotes Activation of β3 Integrins

Zhongji Liao[a], Alexandre R. Gingras[a], Frederic Lagarrigue[a], Mark H. Ginsberg[a], and Sanford J. Shattil[a]

[a]Department of Medicine, University of California, San Diego, La Jolla, CA 92093

To whom correspondence may be addressed. Email: zhliao@health.ucsd.edu or sshattil@health.ucsd.edu.

This PDF file includes:

Figures S1 to S7
Legends for Figures S1 to S7

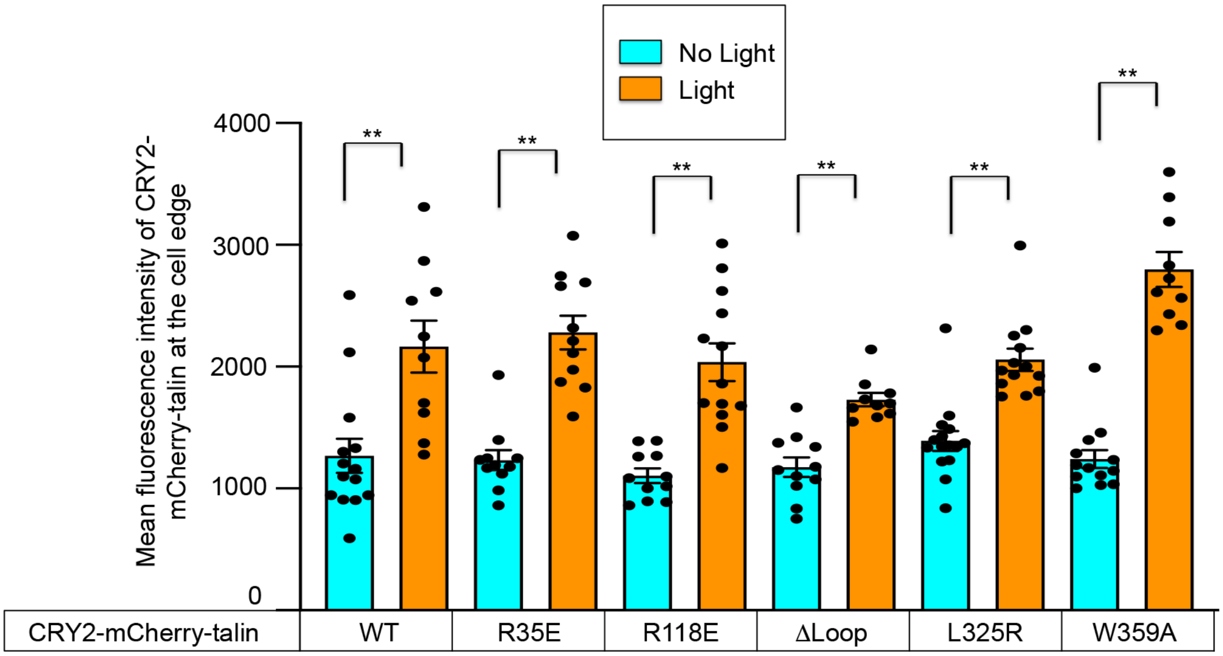

Figure S1. Optogenetic recruitment of talin to the plasma membrane in A5 cells expressing various talin mutants. CIBN-GFP-CAAX was co-expressed in A5 cells with either CRY2-mCherry-wild-type talin or one of the indicated CRY2-mCherry talin mutants. Cells were trypsinized and resuspended before exposure to pulsed blue light for 30 minutes with a frequency of 1 s each 75 seconds. CIBN-GFP-CAAX and CRY2-mCherry-talin in fixed cells were visualized by confocal imaging. Since CIBN-GFP-CAAX was located at the cell edge in the suspended cells, recruitment of CRY2-mCherry-talin by CIBN-GFP-CAAX was quantified as changes of mCherry intensity at cell edges. mCherry intensities were measured on subareas of cell edges that were GFP-positive. Increased fluorescence intensity of mCherry was observed at cell edges in response to blue light. Data represent means ± SEM from 10-15 cells in each of two independent experiments (double asterisk, $P < 0.01$).

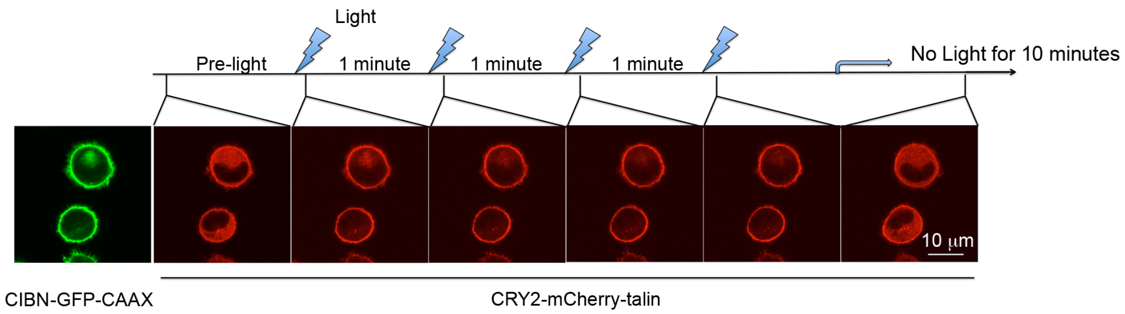

Figure S2. Real time-lapse images of suspended A5 CHO cells expressing CRY2-mCherry-talin and CIBN-GFP-CAAX before and after illumination by 450 nm blue light for the indicated times. Note the rapid optogenetic recruitment of CRY2-mCherry-talin to the plasma membrane and its reversal upon removal of illumination. Scale bar: 10 μm.

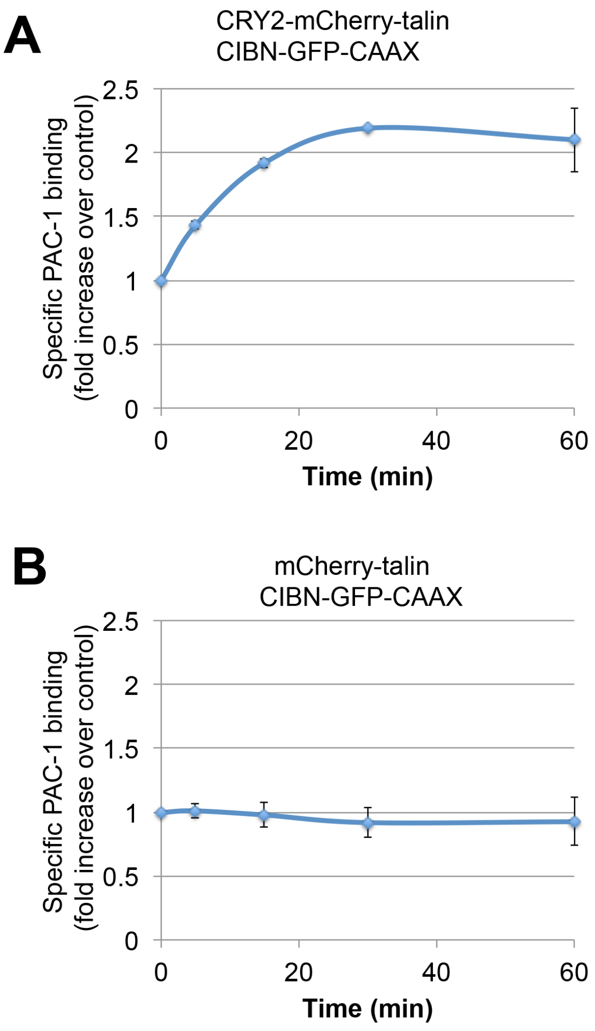

Figure S3. Time course of αIIbβ3 activation (specific PAC-1 binding) in response to blue light in A5 CHO cells expressing CIBN-GFP-CAAX and either CRY2-mCherry-talin (**A**) or mCherry-talin (**B**). Cells in suspension were incubated with PAC-1 and illuminated with blue light for the indicated periods of time, followed by fixation with 3.7% formaldehyde and analysis by flow cytometry. Data represents specific PAC-1 binding normalized to binding observed with cells at zero time in the dark (means ± SEM of three experiments).

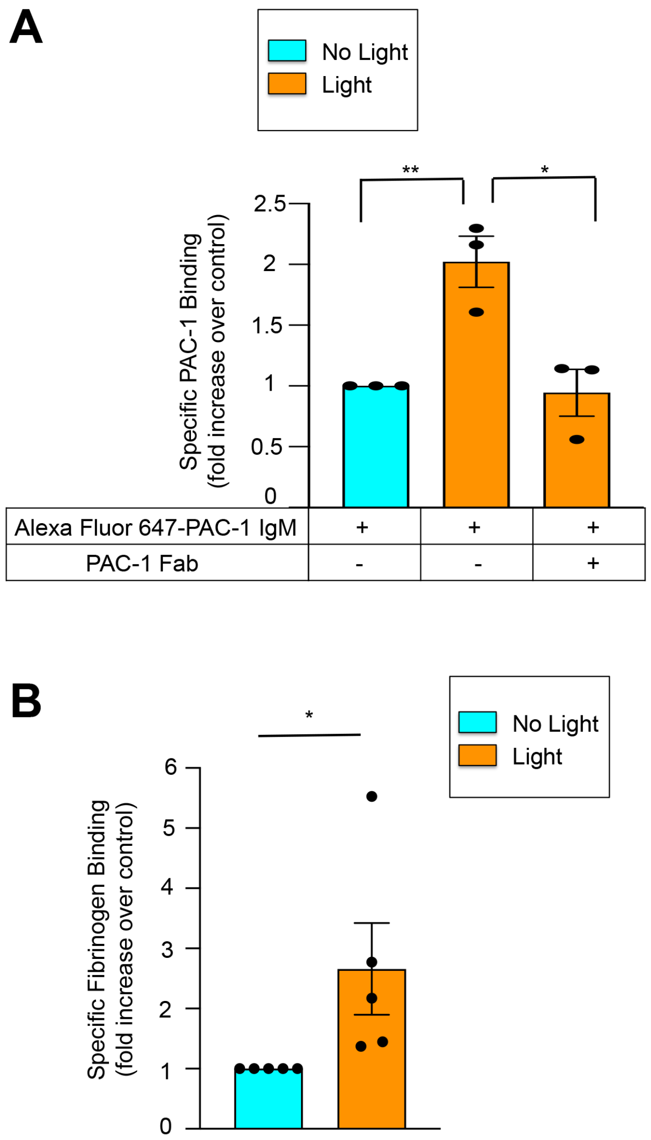

Figure S4. Translocation of full-length talin to the plasma membrane leads to increased αIIbβ3 affinity for fibrinogen. (**A**) Increased PAC-1 binding following optogenetic recruitment of CRY2-mCherry-talin to the plasma membrane was inhibited by PAC-1 Fab, a monovalent Fab fragment of PAC-1. Data represent means ± SEM of three experiments (double asterisk, P < 0.01; asterisk, P < 0.05). (**B**) Optogenetic recruitment of talin to the plasma membrane promoted fibrinogen binding in A5 cells expressing CRY2-mCherry-talin and CIBN-GFP-CAAX. Specific fibrinogen binding was measured by flow cytometry as described in Experimental Procedures. Data represent means ± SEM of five experiments (asterisk, *P*<0.05).

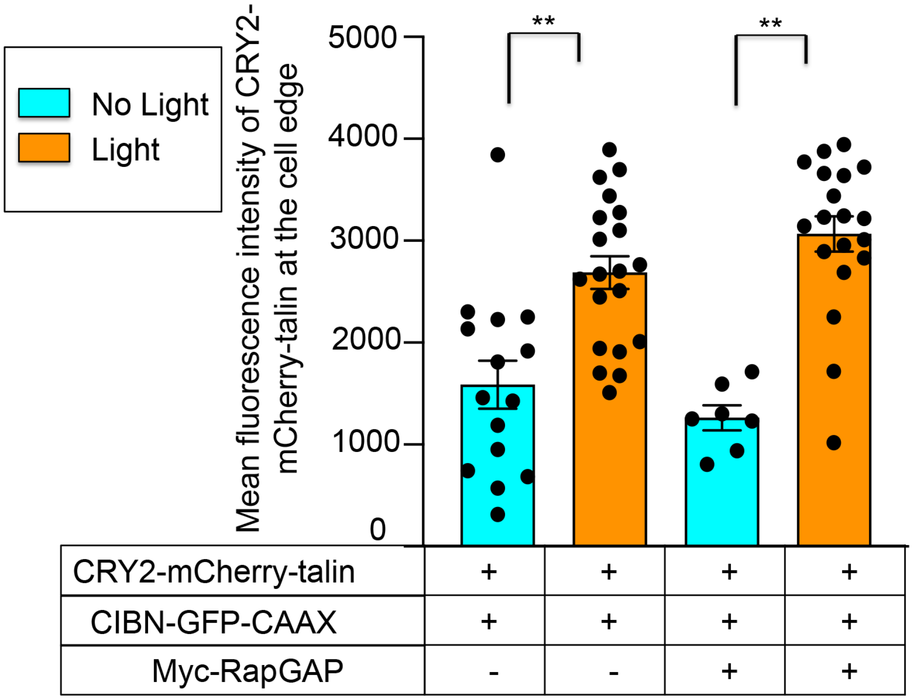

Figure S5. RapGAP does not affect optogenetic recruitment of talin to the plasma membrane in A5 cells. Lentivirus encoding Myc-tagged RapGAP was transduced into A5 cells stably expressing CRY2-mCherry-talin and CIBN-GFP-CAAX. Cells were trypsinized and resuspended before exposure to pulsed blue light for 30 minutes with a frequency of 1 s each 75 seconds. CIBN-GFP-CAAX and CRY2-mCherry-talin in fixed cells were visualized by confocal imaging. Recruitment of CRY2-mCherry-talin by CIBN-GFP-CAAX was quantified as described in Fig. S1. Data represent means ± SEM from 7-20 cells in each of two independent experiments (double asterisk, $P < 0.01$).

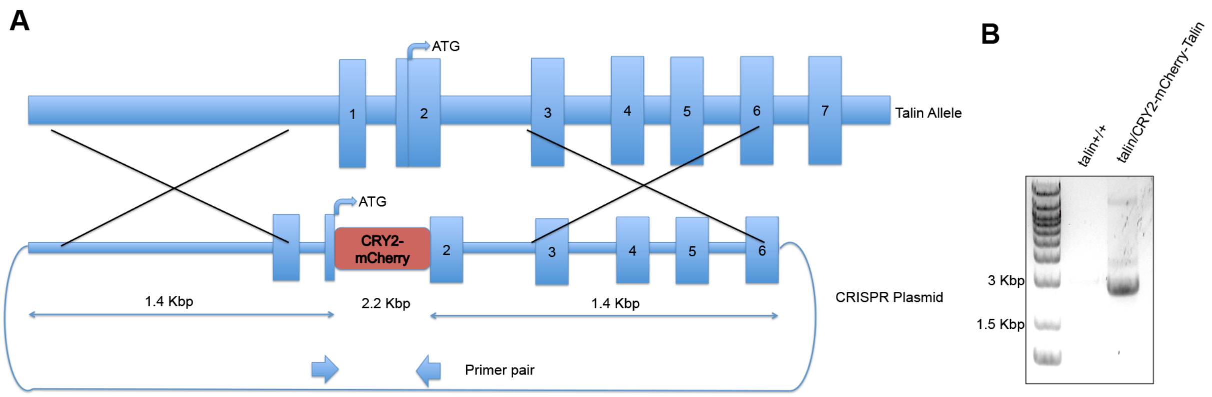

Figure S6. Expression of CRY2-mCherry-talin in immortalized murine lung endothelial cells following CRISPR-Cas9-based homology-directed repair. (**A**) Schematic of the CRISPR-Cas9 strategy to "tag" endogenous talin with CRY2-mCherry as described in Experimental Procedures. (**B**) A pair of primers that flank the CRY2-mCherry sequence was used to amplify and verify its insertion by PCR using genomic DNA as template. Primers covered portions from both CRY2-mCherry cDNA and exon 2 resulting in a PCR product size of ~2.3 Kbp.

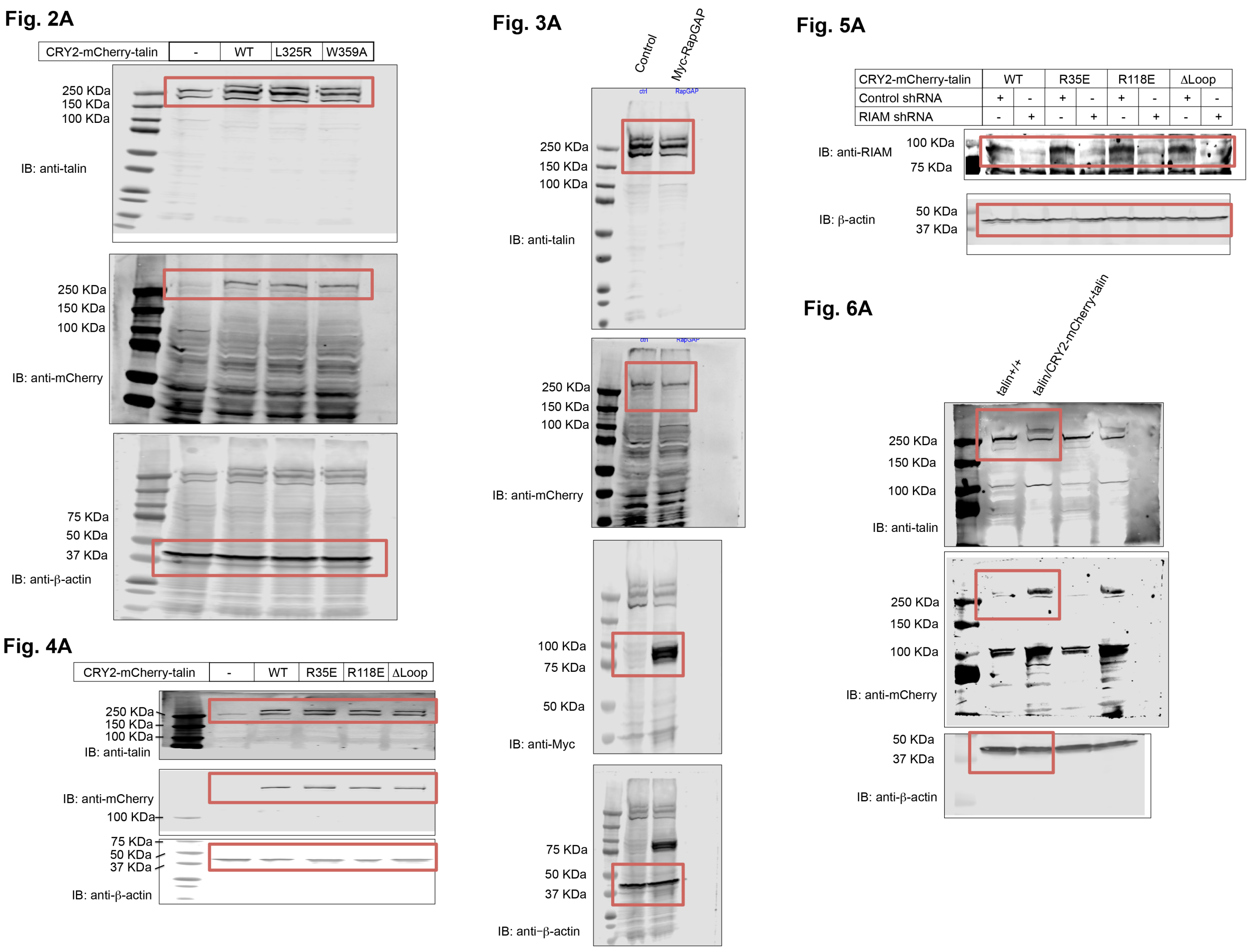

Figure S7. Uncropped images of western blots. The areas used in the indicated figures are shown within red boxes. A protein ladder is located on the far left of each gel. The molecular weights of the protein markers are labeled.
